# Reply to: Comment on: Fetal femur length and risk of diabetes in adolescence: a prospective cohort study

**DOI:** 10.1186/s41182-024-00646-9

**Published:** 2024-10-30

**Authors:** Urme Binte Sayeed

**Affiliations:** Graduate School of Comprehensive Human Sciences, University of Tsukuba, 1-1-1 Tennodai, Tsukuba, Ibaraki 305-8575 Japan

We sincerely appreciate the valuable insights provided by Malik Olatunde Oduoye and others regarding our study, “Fetal femur length and risk of diabetes in adolescence: a prospective cohort study” [1]. In this response, we aim to address several of their comments.

Firstly, the authors expressed concern about the role of gestational diabetes mellitus (GDM) as a predisposing factor for diabetes in adolescents and the lack of the information in our study. We acknowledge that maternal GDM is a significant factor in the development of diabetes in offspring. However, due to data limitations, we could not include the variable in the analyses. Our longitudinal study was embedded in a population-based food and nutrition trial (MINIMat trial) among pregnant women from 2002 to 2003 [2], during which GDM was not well-explored among Bangladeshi women, particularly in rural areas. A study conducted in 2005 highlighted that GDM was largely underexplored in the Bangladeshi women and that there was no consensus on its diagnostic criteria at that period [3].

Secondly, the authors mentioned the importance of genetic factors in developing diabetes among children and adolescents. We appreciate this observation and acknowledge that this aspect was mentioned in the limitations of our study.

Thirdly, Oduoye et al. raised the issue of chronic inflammation potentially being a causative agent for Type 2 diabetes mellitus (T2DM) and suggested that certain inflammatory markers could serve as prognostic indicators. The primary objective of our study was to investigate the association between restricted fetal femur growth and the risk of T2DM in adolescents [1]. Consequently, we did not examine the role of inflammatory agents in this research. However, we recommend further studies considering the causative inflammatory agents in developing T2DM in children and adolescents.

Finally, the authors noted the lack of information regarding physical activity and dietary habits among adolescents, which could contribute to the risk of T2DM. We acknowledge that these factors are indeed significant. Due to challenges in collecting comprehensive data on adolescents’ diets and physical activity, we utilized BMI as a surrogate measure to control the effect of these factors in the models.

In conclusion, our study does have several limitations, particularly related to data availability, which challenge its interpretation. We hope that our explanations have adequately addressed the authors’ concerns. We recommend future prospective cohort studies to mitigate these limitations and enhance our understanding. Thank you once again to our colleagues for their interest in our research.

## Data Availability

The data relating to this study are available upon reasonable request.
